# Supplementary material for: Autoinflammation in Syndromic Hidradenitis Suppurativa: The Role of AIM2
Source: Vaccines (Basel). 2023 Jan 11;11(1):162. doi: 10.3390/vaccines11010162 (PMC9862365; doi:10.3390/vaccines11010162)
Supplement: Supplementary file 1 [file vaccines-11-00162-s001.zip › vaccines-2125651-supplementary.pdf]

Supplementary Table S1: List of primers used for PCR amplification and sequencing

Name	Sequence
AIM2-Ex1-F*	GAATCAAGCCCTCAAGCTCTG
AIM2-Ex1-seqF	CTTTGAGTGCCTGCACTCCTC
AIM2-Ex1-seqR	TTCTGGTATAAACAAGCACAC
AIM2-Ex1-R*	TCTCAGAGGTGTGCACAGCAT
AIM2-Ex2-F*	TGAGAATTCCAGAGGTGTTGT
AIM2-Ex2-R*	ACAAACACTTCCTAGAAGCGA
AIM2-Ex3-F*	GTCTCACTGTGTCATCCAAGC
AIM2-Ex3-R*	ATCTCTGATGTGACCCAGAGC
AIM2-Ex4-F*	TTCCACCTATCTTCTGCTGG
AIM2-Ex4-R*	GGCATGTTTACTCTGCAGCT
AIM2-Ex5/6-F*	GCACAGGTAAAATCTGTGTTG
AIM2-Ex5/6-seqR	TCCTCAGTTCTGTGAGATGAA
AIM2-Ex5/6-R*	CTCCTAATTCTTCCCTCTTGT

* Oligonucleotides used for PCR amplification
